# Resource utilization and treatment costs of patients with severe hemophilia A: Real‐world data from the ATHNdataset

**DOI:** 10.1002/jha2.412

**Published:** 2022-03-27

**Authors:** Michael Recht, Chunla He, Er. Chen, Dunlei Cheng, Paul Solari, David Hinds

**Affiliations:** ^1^ American Thrombosis and Hemostasis Network (ATHN) Rochester New York USA; ^2^ BioMarin Pharmaceutical, Inc. (BioMarin) Novato CA USA

**Keywords:** clinical burden, economic burden, hemophilia A, prophylaxis treatment, rare diseases

## Abstract

Hemophilia A is characterized by unpredictable spontaneous bleeds and chronic comorbidities. However, limited data exists at the national level into detailed management patterns related to patient clinical characteristics, representative real‐world dosing and treatment frequency, and costs. To assess and characterize the US severe hemophilia A (SHA) population, including subgroups of patients, in terms of clinical and demographic characteristics, healthcare resource utilization received at hemophilia treatment centers (HTCs), and projected annual costs of treatment utilizing data from the ATHNdataset of the American Thrombosis and Hemostasis Network (ATHN). Adult male people with SHA (PwSHA) (FVIII < 1%) were identified in the ATHNdataset between January 2013 and September 2019. This retrospective cohort study described patients’ demographic and clinical characteristics, clinical history, as well as the HTC‐related health resource utilization (HRU), treatment utilization, and projected annual treatment costs of US PwSHA received over the most recent year. Results are reported for the overall population and for three mutually exclusive subpopulations of patients: PwSHA with a history of and/or current inhibitors, PwSHA without a history of inhibitors but with (or a history of) one or more transfusion‐transmitted infections (hepatitis B virus [HBV], hepatitis C virus [HCV], or human immunodeficiency virus [HIV]), and PwSHA without a history of inhibitors or of transfusion‐transmitted infections (HBV, HCV, or HIV). Of the overall PwSHA cohort (*N* = 3677), there was a high prevalence of HCV (24.1%) and HIV (13.7%), while the prevalence of HBV (4.9%) was lower. Note that 20.5% of PwSHA overall currently or ever had FVIII inhibitors. On average, PwSHA had 2.8 total HTC visits per year, including 0.9 comprehensive care visits, 1.1 telephone contact visits, 0.5 office visits, and 0.1 surgeries or other procedures. However, 23.3% of PwSHA were not seen at an HTC, and 33.8% of PwSHA did not have a comprehensive care visit during their most recent year of data. HTC‐related HRU was similar between the overall cohort and across the patient subpopulations, although PwSHA and inhibitors had more frequent HTC visits (a mean of 3.6 visits annually vs. 2.5–2.8 in the other groups). Using reported treatment frequency and dosing, estimated mean annual hemophilia treatment costs varied by treatment and across the three subpopulations: extended half‐life factor product ($893,609–934,301 by subpopulation), standard half‐life factor product ($798,700–930,812), plasma‐derived factor product ($613,220–801,061), and non‐factor product treatment ($765,289—833,240). This study summarized recent sociodemographic and clinical characteristics, HTC‐related HRU, and HA treatments and projected costs among adult PwSHA, including among key subpopulations of PwSHA. PwSHA experience substantial clinical and resource burden on a chronic basis, despite the care coordination efforts of ATHN‐affiliated HTCs. These findings motivate further exploration of the drivers of resource utilization, observed differences across subpopulations and other disparities, and ongoing monitoring of clinical and treatment burden in the face of an evolving care landscape.

## 1 INTRODUCTION

Hemophilia A (HA) is an X‐linked coagulation factor disorder primarily affecting males and caused by a deficiency of 40% or less than normal levels of the blood clotting factor VIII (FVIII) [1]. Because of insufficient endogenous levels of FVIII needed for normal blood clotting, patients with HA experience bleeds of varying severity (from clinically silent to life‐threatening) occurring spontaneously or in response to minor injury [2]. HA is present in approximately one in 5000 live male births based on data from US hemophilia treatment centers (HTCs), or 400 infants newly diagnosed with HA in the United States annually [1, 2, 3, 4, 5], with global estimates of the birth prevalence of approximately one in 4000 live male births based on a recent meta‐analysis [6]. Approximately, 22,000 PwHA have been treated at a US HTC between 2012 and 2021 [7].

Half to two‐thirds of people diagnosed with HA have severe HA (SHA; i.e., FVIII activity < 1 percent of normal) and are at high risk of painful and potentially dangerous bleeding events, as well as joint and organ damage, need for surgeries, chronic arthropathy and other long‐term complications, as well as lowered quality of life [2, 8, 9]. Routine management of HA involves prophylactic and acute (i.e., on‐demand) administration of FVIII to levels permitting normal clotting [10]. FVIII replacement is effective in reducing bleeding and negative outcomes, permitting a more active life [11, 12]. However, a major and common complication of FVIII replacement is the development of inhibitor antibodies in response to factor infusions, greatly reducing the ability of prophylactic or on‐demand therapy to prevent or stop bleeding and increasing mortality risk [13]. In addition, treatment guidelines highlight additional considerations for the management of patients who are inhibitor‐positive and have a transfusion‐transmitted infection (e.g., positive for hepatitis B virus [HBV], hepatitis C virus [HCV], or human immunodeficiency virus [HIV]) patients [10]. The introduction of emicizumab in recent years, a recombinant, humanized, and bispecific monoclonal antibody, provided another treatment option for patients with or without FVIII inhibitors [10]. Understanding and describing the current SHA population is key to understanding the current burden of illness of SHA, and best approaches for management of the disease, as treatments evolve.

The economic burdens and impact on quality of life of FVIII replacement are also substantial due to the high costs of therapy, required treatment intensity and frequency, and monitoring over the span of a patient's entire life [14, 15]. Recent estimates from registry studies and insurance claims analyses of the annual payer costs for patients receiving prophylactic therapies have ranged from $256,426 to 753,480 per year [16, 17, 18, 19, 20]. Based on Medicare spending in 2010, hemophilia treatments were the most costly drug on average per beneficiary [21]. Furthermore, some subpopulations of patients with HA, such as those with inhibitors or transfusion‐transmitted infections, experience additional complications, worse clinical outcomes, and greater economic burden as FVIII therapy have reduced effectiveness. Finally, even with optimal management, breakthrough bleeds, lifestyle restriction, and high healthcare resource utilization (HRU) are still expected for patients with HA [22, 23].

The treatment landscape for HA continues to evolve in response to the burden of treatment and the disease, with several new therapies in clinical development. For example, prophylactic FVIII replacement therapies include both standard half‐life (SHL) and extended half‐life formulations (EHL), with EHL formulations providing longer prophylactic benefits and decreased infusion frequency at higher up‐front cost [16, 24]. In a 2020 real‐world study by Yan et al. of 240 patients with HA (191 with SHA), EHL FVIII treatments demonstrated comparable efficacy as SHL treatments with regard to annual bleed rates and the proportion of patients experiencing no bleeds, despite reduced dosing frequency [25]. Additionally, nonfactor substitution product treatments such as emicizumab and hemostasis rebalancing therapies have been developed, featuring decreased treatment frequency, more convenient subcutaneous dosing, and the potential for improved outcomes relative to traditional intravenous FVIII prophylaxis therapies in patients both with and without inhibitors [26, 27, 28]. Gene therapies and other new breakthrough therapies being developed have the potential to further reduce treatment burden and improve patient outcomes for a subset of patients with HA.

This study aimed to characterize the US SHA population including subgroups of patients regarding clinical and demographic characteristics, HTC‐related HRU, treatments, and projected annual costs of treatment utilizing data from the ATHNdataset derived from HTCs in the American Thrombosis and Hemostasis Network (ATHN). Along with the overall ATHN population with SHA, three mutually exclusive subpopulations, which may impact HRU, were described based on presence of inhibitors, transfusion‐transmitted infections, without inhibitors, and no history of inhibitors or transfusion‐transmitted infections.

## 2 METHODS

### 2.1 Study overview

This retrospective cohort study of people with SHA (PwSHA) described the demographic and clinical characteristics, clinical history, and the HTC‐related HRU, treatment utilization, and projected annual treatment costs for prophylaxis with FVIII replacement or nonfactor therapies adult males with SHA treated at one of 146 ATHN‐affiliated HTCs in the US. Data from the ATHNdataset were included for patients active in the database from January 1, 2013 to September 30, 2019 (study period). The analysis period for each PwSHA was defined as the date of first activity in the ATHNdataset during the study period and lasted up to and including the date of the last activity in the dataset.

### 2.2 Data source

The ATHNdataset is a HIPAA‐compliant de‐identified patient health dataset containing data from individuals with bleeding and clotting disorders receiving care through the US HTC Network. Individuals consent or opt‐in to contribute their data in order to help establish a better understanding of bleeding and clotting disorders such as the complications of these disorders, the social and economic costs, and the effectiveness of treatments and interventions. The ATHNdataset contains data recorded in the electronic medical records at individual HTCs and entered into the ATHNdataset, primarily reflecting health care interactions at the HTC. With data contributed by over 40,000 individuals, approximately 13,000 of whom have HA, the ATHNdataset is the largest source of health data of people with hemophilia in the world [29].

### 2.3 Study population

Adult (≥18 years) male PwSHA observed in the ATHNdataset any time between January 1, 2013 and September 30, 2019 were included in the study cohort. For this analysis, patients had to have at least 1 year of activity in the ATHNdataset. Age was based on the most recent medical encounter in the ATHNdataset. SHA was identified based on FVIII activity < 1% of normal as recorded in the ATHNdataset. Individuals with any bleeding disorder other than HA were excluded (i.e., hemophilia B, Von Willebrand factor deficiencies, other bleeding conditions), even if those patients also had HA.

The overall population was divided into three mutually exclusive subpopulations of patients based on the potential for varying resource use and treatment costs: 1) PwSHA with a history of and/or current inhibitors, 2) PwSHA without a history of inhibitors but with (or a history of) transfusion‐transmitted infections (HBV, HCV, or HIV), and 3) PwSHA without inhibitors or a history of three transfusion‐transmitted infections (HBV, HCV, or HIV).

### 2.4 Key variables and analyses

Sociodemographic and clinical characteristics were described based on the most recent activity in the ATHNdataset including age, weight, height, body mass index (BMI), geographic region, self‐reported race/ethnicity, and education level. Clinical history was also summarized including age at first bleed, age at first treatment with FVIII, age at ATHN enrollment, recorded target joints, and history of key comorbidities (arthropathy, HBV, HCV, HIV, and inhibitor‐positive status) as recorded in the ATHNdataset.

HTC‐related HRU, including utilization of treatments and treatment regimen, along with projected annual treatment costs as recorded in the ATHNdataset were described. HTC‐HRU were derived from the medical records at ATHN‐affiliated HTCs. HTC‐related HRU at ATHN‐affiliated HTCs was estimated based on the most recent year of data for each PwSHA (2018‐2019) including any HTC visits, comprehensive care visits, telephone contact visits, office visits, recorded surgery/procedure (may have occurred outside of HTC), as well as joint replacements. HA‐related treatment with factor and non‐factor product therapies were described based on a patients’ most recent activity in the ATHNdataset. Treatment regimen and utilization of treatments (by class) were summarized for SHL, EHL, and plasma‐derived FVIII concentrates, and non‐factor product therapies. Description of treatment regimens included mean dose (i.e., per kg or per mg), average frequency (days/treatment), and all‐cause discontinuation rate.

While cost data are not recorded in the ATHNdataset, projected annual costs by class were estimated based on treatment‐specific utilization and treatment regimen characteristics. Mean annual costs of prophylaxis were projected based on applying wholesale acquisition cost (WAC) prices for specific treatment [30] to the recorded dose, dose frequency for a patient/product, and aggregated for treatment class (EHL, SHL, plasma‐derived, and non‐factor products). Costs were estimated based on 2020 US dollar (USD) costs for a product.

Data were summarized using descriptive statistics in SAS 9.4. Continuous variables were summarized using N, mean, SD, median, and interquartile range (IQR). Categorical variables were summarized using frequency and percentage. Where applicable, missing variables were presented as missing, with no imputation for missing data. Unknown or missing data indicates that the data was not reported to the ATHNdataset during the reporting period [29].

## 3 RESULTS

### 3.1 Sample selection

**FIGURE 1:**
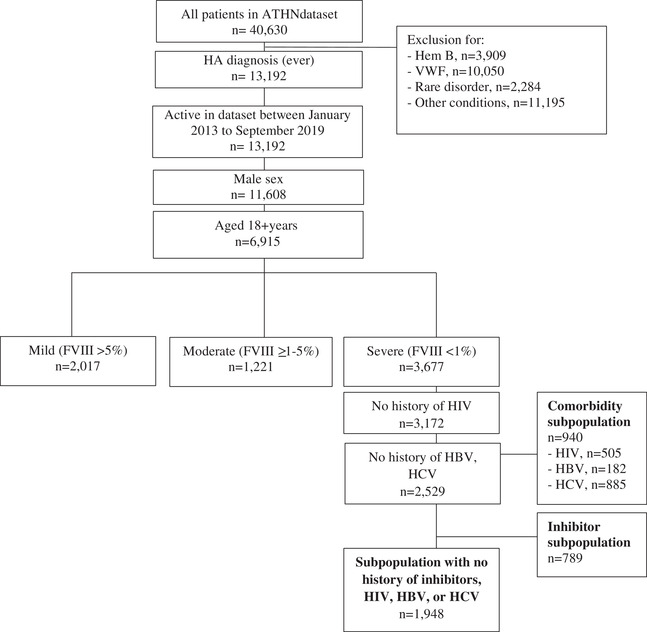
Sample selection and classification. HBV, hepatitis B virus; HCV, hepatitis C virus; Hem B, hemophilia B; HIV, human immunodeficiency virus; IQR, interquartile range; SD, standard deviation; VWF, Von Willebrand factor deficiencies

The inclusion and exclusion criteria and the final SHA sample counts by subpopulation are presented in Figure 1. A total of 6915 adult males with HA were active in the ATHNdataset between 2013 and 2019 including 3677 PwSHA (FVIII < 1% of normal). The overall sample of PwSHA was further subdivided into three mutually exclusive subpopulations: (1) those with a history of inhibitors (*n* = 789), (2) those with no history of inhibitors but with a transfusion‐transmitted infection (i.e., HIV, HBV, or HCV) but no history of inhibitors (*n* = 940), and (3) those with no history of inhibitors or a transfusion‐transmitted infection (*n* = 1948). The mean observation time in the database was 6 (median: 6.6; IQR: 4.5–8.0) years for the overall cohort.

### 3.2 Sociodemographic characteristics of PwSHA

Patients in the prespecified subpopulations were generally similar regarding demographic characteristics, with the exception of age (Table 1). The overall cohort represented a younger adult population with >50% being <35 years of age (34.0% were 18–26 years old, 30.4% were 27–35 years old, 32.4% were 35–65 years old, and 3.1% were over 65 years old). PwSHA and a history of transfusion‐transmitted infections were older than the overall population and other prespecified subpopulations, with 83.4% of this subgroup aged older than 35 years compared with 35.5% of the overall cohort. Patients were treated at ATHN‐affiliated HTCs throughout the US and with the majority of patients being White, non‐Hispanic. The majority of patients across all cohorts were White (70.1%–79.8%), while the subgroup with a history of inhibitors was observed to have a higher proportion of Black or African American patients (20.3% vs. 13.3% and 14.2% in the other subpopulations). The proportions of patients of other races, including Native Hawaiian or other Pacific Islander, American Indian, or Alaska Native, and mixed race patients, were small (combined 2.2%); data on race was missing for 1.8% of patients. The majority of patients were non‐Hispanic (86.6%), although Hispanic patients were represented in the study sample (13%). Education level was generally consistent across subpopulations, although level spanned the education spectrum with ∼50% of the population having a college degree or higher.

**TABLE 1:** Sociodemographic and clinical characteristics of the American Thrombosis and Hemostasis Network (ATHN) adult hemophilia A (HA) population, by severity and by subpopulation

		Subpopulation		
	PwSHA	PwSHA and inhibitors	PwSHA, no inhibitors, and HBV/HCV/HIV	PwSHA, no inhibitors, and no HBV/HCV/HIV
	(*n* = 3677)	(*n* = 789)	(*n* = 940)	(*n* = 1948)
**Current age, *n* (%)**				
18–26 years	1251 (34.0%)	313 (39.7%)	21 (2.2%)	917 (47.1%)
27–35 years	1118 (30.4%)	241 (30.5%)	135 (14.4%)	742 (38.1%)
36–65 years	1193 (32.4%)	213 (27.0%)	712 (75.7%)	268 (13.8%)
65+ years	115 (3.1%)	22 (2.8%)	72 (7.7%)	21 (1.1%)
**Weight, mean, kg (SD)** ^a^	84.5 (22.5)	84.0 (22.9)	84.9 (21.2)	84.6 (22.9)
Missing weight	107 (2.9%)	16 (2.0%)	25 (2.7%)	66 (3.4%)
**Height, mean, meters (SD)** **^a^**	1.75 (0.09)	1.75 (0.09)	1.76 (0.09)	1.76 (0.09)
Missing height	480 (13.1%)	100 (12.7%)	137 (14.6%)	243 (12.5%)
**Body mass index, mean, kg/m^2^ (SD)** ^a^	27.3 (0.8)	27.4 (13.0)	27.4 (6.3)	27.3 (7.0)
Missing weight or height	520 (14.1%)	108 (13.7%)	146 (15.5%)	266 (13.7%)
**Region, *n* (%)**				
Great Lakes	439 (11.9%)	93 (11.8%)	110 (11.7%)	236 (12.1%)
Great Plains	438 (11.9%)	84 (10.6%)	122 (13.0%)	232 (11.9%)
Mid‐Atlantic	414 (11.3%)	76 (9.6%)	90 (9.6%)	248 (12.7%)
Mountain States	462 (12.6%)	107 (13.6%)	110 (11.7%)	245 (12.6%)
New England	488 (13.3%)	95 (12.0%)	150 (16.0%)	243 (12.5%)
Northern States	325 (8.8%)	58 (7.4%)	95 (10.1%)	172 (8.8%)
Southeast	671 (18.2%)	177 (22.4%)	168 (17.9%)	326 (16.7%)
Western States	440 (12.0%)	99 (12.5%)	95 (10.1%)	246 (12.6%)
**Race, *n* (%)**				
White	2794 (76.0%)	553 (70.1%)	750 (79.8%)	1491 (76.5%)
Black or African American	561 (15.3%)	160 (20.3%)	125 (13.3%)	276 (14.2%)
Asian	177 (4.8%)	37 (4.7%)	33 (3.5%)	107 (5.5%)
Other/Missing^b^	145 (4.0%)	39 (4.9%)	32 (3.5%)	74 (3.7%)
**Ethnicity, *n* (%)**				
Hispanic	477 (13.0%)	123 (15.6%)	75 (8.0%)	279 (14.3%)
**Education level, *n* (%)**				
Pre‐elementary	12 (0.3%)	3 (0.4%)	0 (0%)	9 (0.5%)
Primary or secondary	1226 (33.3%)	255 (32.3%)	261 (27.8%)	710 (36.4%)
GED or equivalent	270 (7.3%)	60 (7.6%)	45 (4.8%)	165 (8.5%)
Technical school	162 (4.4%)	39 (4.9%)	63 (6.7%)	60 (3.1%)
College	1377 (37.4%)	310 (39.3%)	343 (36.5%)	724 (37.2%)
Advanced degree	244 (6.6%)	45 (5.7%)	99 (10.5%)	100 (5.1%)
Other/missing	386 (10.5%)	77 (7.7%)	129 (13.7%)	180 (9.3%)
**Age at ATHN enrollment, years**				
Mean (SD)	28.2 (13.0)	26.8 (12.8)	40.2 (11.5)	22.9 (9.5)
Median (IQR)	25.1 (18.6–34.5)	22.9 (17.8–32.3)	37.7 (31.3–48.3)	21.3 (16.6–27.0)
**Age at first bleed, mean years (SD)**	1.1 (3.2)	1.0 (2.5)	1.3 (3.8)	1.0 (3.2)
Missing age at first bleed	2666 (72.5%)	548 (69.5%)	732 (77.9%)	1386 (71.1%)
**Age at first FVIII treatment, mean years (SD)**	1.6 (4.2)	2.1 (5.6)	1.5 (3.5)	1.4 (3.7)
Missing age at first treatment with FVIII	3101 (84.3%)	650 (82.4%)	841 (89.5%)	1610 (82.6%)
**Target joint, *n* (%)**				
Ankle	413 (11.2%)	94 (11.9%)	132 (14.0%)	187 (9.6%)
Elbow	329 (8.9%)	72 (9.1%)	103 (11.0%)	154 (7.9%)
Knee	232 (6.3%)	63 (8.0%)	77 (8.2%)	92 (4.7%)
Shoulder	58 (1.6%)	13 (1.6%)	26 (2.8%)	19 (1.0%)
Other joints	26 (0.7%)	8 (1.0%)	10 (1.1%)	8 (0.4%)
Not reported	3094 (84.1%)	653 (82.8%)	772 (82.1%)	1669 (85.7%)
**Comorbidity history, *n* (%)**				
Ever had arthropathy	400 (10.9%)	92 (11.7%)	151 (16.1%)	157 (8.1%)
Ever had HBV	182 (4.9%)	39 (4.9%)	143 (15.2%)	0 (0.0%)
Ever had HCV	885 (24.1%)	167 (21.2%)	718 (76.4%)	0 (0.0%)
Ever had HIV	505 (13.7%)	55 (7.0%)	450 (47.9%)	0 (0.0%)
Ever had inhibitors	789 (21.5%)	789 (100.0%)	0 (0.0%)	0 (0.0%)

Abbreviations: GED, general educational development test; HBV, hepatitis B virus; HCV, hepatitis C virus; HIV, human immunodeficiency virus; IQR, interquartile range; SD, standard deviation; SHA, severe hemophilia A.

^a^ Sociodemographic and clinical characteristics were defined based on the most recent record observed.

^b^ Other/missing includes the categories of Native Hawaiian or Pacific Islander, American Indian or Alaska Native, mixed race, and missing race.

### 3.3 Clinical characteristics of PwSHA

#### 3.3.1 Comorbid conditions

The overall cohort of PwSHA had high prevalence of HCV (24.1%) and HIV (13.7%), while the prevalence of HBV was lower at 4.9% (see Table 1). A total of 20.5% of patients in the overall cohort currently or ever had FVIII inhibitors. Prevalence of comorbidities within subgroups was related to the prespecified subgroup definitions. Among the subgroup of patients with transfusion‐transmitted infections without inhibitors, HCV was most common (76.4%), while approximately half (47.9%) of this subgroup was HIV‐positive.

The overall prevalence of arthropathy recorded for the cohort was 10.9%. Patients with transfusion‐transmitted infections without inhibitors had the highest rate of a history of arthropathy (16.1%) across the three subpopulations, following by patients with inhibitors (11.7%). Conversely, the cohort with no history of inhibitors and no history of a transfusion‐transmitted infection had a lower rate of a history of arthropathy (8.1%). The population on average was overweight, with BMIs generally similar across the cohorts of approximately 27 kg/m^2^.

#### 3.3.2 Hemophilia A history

On average, the cohort was 28.2 years old when they first enrolled in ATHN through an affiliated HTC, with a higher age at enrollment for the subpopulation of patients with transfusion‐transmitted infections (mean 40.2 years old) compared with other patients without inhibitors (22.9 years) and patients with a history of inhibitors (26.8 years). Target joints were recorded for approximately 15% of the population, with the ankle being the most commonly reported target joint (11.2%) followed by the elbow (8.9%), knee (6.3%), shoulder (1.6%) and other joints (0.7%).

### 3.4 Annual HTC‐related HRU

Annual HTC‐related HRU among the overall study and subgroups is presented in Table 2. In general, comparable HRU was observed among the overall cohort and across the patient subgroups, although patients with inhibitors had more frequent visits (a mean of 3.6 visits annually vs. 2.5–2.8 in the other groups). On average, PwSHA had 2.8 total HTC visits (median: 1.0) per year with of 0.9 (1.0) comprehensive care visits, 1.1 (3.3) telephone contact visits, 0.5 (0.0) office visits, and 0.1 (0.0) surgeries or procedures. Although on average, PwSHA were being seen at an HTC multiple times per year and having a comprehensive care visit annually, approximately 25% of the overall population did not have an HTC visit annually, and 33% did not have a comprehensive care visit, which allows for the multidisciplinary HTC team to respond to patient needs and identifying gaps in care [31].

**TABLE 2:** HTC‐related health resource utilization (HRU) over 1 year in the American Thrombosis and Hemostasis Network (ATHN) adult SHA population, overall and by subpopulation

		Subpopulation		
	All PwSHA	PwSHA and inhibitors	PwSHA, no inhibitors, and HBV/HCV/HIV	PwSHA, no inhibitors, and no HBV/HCV/HIV
	(*n* = 3677)	(*n* = 789)	(*n* = 940)	(*n* = 1948)
**HTC visits (any)** **^a^**				
Mean (SD)	2.8 (5.2)	3.6 (6.7)	2.8 (5.5)	2.5 (4.2)
Median (IQR)	1.0 (1.0–3.0)	1.0 (1.0–3.0)	1.0 (1.0‐2.0)	1.0 (0.5–3.0)
≥1	2813 (76.5%)	622 (78.8%)	730 (77.7%)	1461 (75.0%)
2	553 (15.0%)	125 (15.8%)	143 (15.2%)	285 (14.6%)
>2	982 (26.7%)	260 (33.0%)	233 (24.8%)	489 (25.1%)
**Comprehensive care visit**				
Mean (SD)	0.9 (0.9)	1.0 (1.0)	0.9 (0.8)	0.9 (0.8)
Median (IQR)	1.0 (0.0–1.0)	1.0 (0.0–1.0)	1.0 (0.0–1.0)	1.0 (0.0–1.0)
≥1	2434 (66.2%)	533 (67.6%)	631 (67.1%)	1270 (65.2%)
**Telephone contact visit**				
Mean (SD)	1.1 (3.3)	1.5 (4.1)	1.0 (3.6)	0.9 (2.8)
Median (IQR)	0.0 (0.0–0.0)	0.0 (0.0–0.0)	0.0 (0.0–0.0)	0.0 (0.0–0.0)
≥1	751 (20.4%)	195 (24.7%)	177 (18.8%)	379 (19.5%)
**Office visit**				
Mean (SD)	0.5 (2.0)	0.7 (3.4)	0.5 (1.7)	0.4 (1.4)
Median (IQR)	0.0 (0.0–0.0)	0.0 (0.0–1.0)	0.0 (0.0–0.0)	0.0 (0.0–0.0)
≥1	835 (22.7%)	215 (27.2%)	203 (21.6%)	417 (21.4%)
**Surgery/Procedure (any)**				
Mean (SD)	0.1 (0.3)	0.0 (0.3)	0.1 (0.5)	0.0 (0.2)
Median (IQR)	0.0 (0.0–0.0)	0.0 (0.0–0.0)	0.0 (0.0–0.0)	0.0 (0.0–0.0)
≥1	143 (3.9%)	30 (3.8%)	52 (5.5%)	61 (3.1%)
2	17 (0.5%)	3 (0.4%)	10 (1.1%)	4 (0.2%)
>2	12 (0.3%)	1 (0.1%)	7 (0.7%)	4 (0.2%)
**Joint replacement procedure**				
Mean (SD)	0.0 (0.1)	0.0 (0.1)	0.0 (0.1)	0.0 (0.1)
Median (IQR)	0.0 (0.0–0.0)	0.0 (0.0–0.0)	0.0 (0.0–0.0)	0.0 (0.0–0.0)
≥1	21 (0.6%)	5 (0.6%)	9 (1.0%)	7 (0.4%)

Abbreviations: HA, hemophilia A; HBV, hepatitis B virus; HCV, hepatitis C virus; HIV, human immunodeficiency virus; HTC, hemophilia treatment center; IQR, interquartile range; SD, standard deviation; SHA, severe hemophilia A.

^a^ HTC visits (any) include comprehensive care visits, telephone contact visits, office visits, and any visits associated with surgeries/procedures received at ATHN‐affiliated HTCs.

^b^ Note HA‐related HRU provided by medical providers at facilities other than ATHN‐affiliated HTCs were not observed.

In addition to more visits overall, the patient subgroup with inhibitors had higher rates of all other HTC visit or contact types with the exception of surgeries or procedures. The annual incidence of any surgery/procedure was 3.9% in the overall cohort recorded in the dataset, with low rates of both specific surgeries/procedures (e.g., joint replacement surgeries) as well as patients undergoing multiple surgeries or other procedures.

### 3.5 Treatment characteristics

In the overall cohort, 8.1% used on‐demand treatment, 76.4% used FVIII prophylaxis treatments, 12.1% used nonfactor substitution product treatments, and data concerning treatment type was missing in the remaining 3.4% of the cohort. The distribution of prophylaxis and other (i.e., on‐demand and nonfactor products) HA treatments for the overall population and patient subgroups is presented in Table 3, along with mean dosing and frequency of treatments for each type of prophylaxis (i.e., SHL, EHL, plasma‐derived) and nonfactor product treatments. Among prophylaxis treatments, annual discontinuation rates were highest for patients treated with plasma‐derived products (7.5% in the overall SHA population), followed by SHL product treatment (5.9%), and with EHL product treatment the lowest (2.6%). Observed discontinuation for nonfactor product treatment was very low (0.2% in the overall cohort). Patients with inhibitors had the highest rate of treatment discontinuation (9.0% of SHL and 4.6% of EHL), the most frequent prescribed dosing (2.6 and 3.6 days, respectively), the lowest use of prophylaxis (65.7%), and generally higher average doses. Conversely, patients without inhibitors or transfusion‐transmitted infections had lower rates of discontinuation, slightly less frequent prescribed dosing, and higher use of prophylaxis than in the overall cohort.

**TABLE 3:** Hemophilia A (HA)‐related treatments observed over 1 year in the American Thrombosis and Hemostasis Network (ATHN) adult SHA population, overall and by subpopulation^a^

		Subpopulation		
	All PwSHA	PwSHA and inhibitors	PwSHA, no inhibitors, and HBV/HCV/HIV	PwSHA, no inhibitors, and no HBV/HCV/HIV
	(*n* = 3677)	(*n* = 789)	(*n* = 940)	(*n* = 1948)
**FVIII treatments**				
**On‐demand treatments, *n* (%)**	297 (8.1%)	65 (8.2%)	131 (13.9%)	101 (5.2%)
**Prophylaxis treatments, *n* (%)**	2808 (76.4%)	518 (65.7%)	670 (71.3%)	1620 (83.2%)
**SHL products**				
Mean dose per kg (IU/kg)	39.3	40.3	39.1	39.1
Average frequency (days/treatment)	2.8	2.6	2.8	2.8
Discontinuation rate	5.9%	9.0%	6.1%	5.0%
**EHL products**				
Mean dose per kg (IU/kg)	48.5	50.2	47.9	48.4
Average frequency (days/treatment)	3.8	3.6	3.9	3.8
Discontinuation rate	2.6%	4.6%	1.9%	2.4%
**Plasma‐derived products**				
Mean dose per kg (IU/kg)	46.9	58.8	39.8	42.0
Average frequency (days/treatment)	2.5	2.4	2.7	2.6
Discontinuation rate, %	7.5%	7.9%	7.3%	7.3%
**Non‐factor product treatments, *n* (%)**	445 (12.1%)	178 (22.6%)	96 (10.2%)	171 (8.8%)
Mean dose per mg (mg/kg)	3.0	2.6	3.2	3.5
Average frequency (days/treatment)	11.3	18.3	12.0	11.9
Discontinuation rate	0.2%	0.6%	1.0%	0.0%

Abbreviations: EHL, extended half‐life formulation; HBV, hepatitis B virus; HCV, hepatitis C virus; HIV, human immunodeficiency virus; SHA, severe hemophilia A; SHL, standard half‐life formulation; UI, International Unit.

^a^ Most recent year of data considered for each patient was defined from the most recent encounter.

#### 3.5.1 Estimated annual costs of prophylaxis treatment

The estimated costs of SHL, EHL, plasma‐derived, and nonfactor products, based on observed HRU and treatment pricing assumptions, are presented in Table 4. For the overall cohort and the patient subgroups, the estimated mean annual treatment costs were highest for EHL products (ranging from $893,609 to 934,301 across the subgroups), followed by SHL and plasma‐based treatments. The annual treatment costs with SHL products ranged from $798,700 to 930,812 across cohorts and plasma‐derived costs ranged from $613,220 to 801,061. The estimated mean annual treatment cost for nonfactor substitution products was comparable to that of SHL products ($821,567 vs. 802,746 in the overall population, respectively), with both annual cost estimates being lower than EHL products. One exception was in the subpopulation of PwSHA and inhibitors, where nonfactor products were estimated to have a lower mean annual treatment cost compared to each type of prophylaxis treatment.

**TABLE 4:** Estimated mean treatment costs over 1 year in the American Thrombosis and Hemostasis Network (ATHN) adult SHA population, overall and by subpopulation^a^, ^b^

		Subpopulation		
	All patientswith SHA	PwSHA and inhibitors	PwSHA, no inhibitors, and HBV/HCV/HIV	PwSHA, no inhibitors, and no HBV/HCV/HIV
	(*n* = 3677)	(*n* = 789)	(*n* = 940)	(*n* = 1948)
**Prophylaxis treatment annual cost**				
**SHL products**	$821,567	$930,812	$798,700	$802,017
**EHL products**	$922,449	$934,301	$893,609	$931,370
**Plasma‐derived products**	$685,256	$801,061	$613,220	$640,574
**Nonfactor product treatments annual cost**	$802,746	$765,289	$833,240	$824,668

Abbreviations: EHL, extended half‐life formulation; HBV, hepatitis B virus; HCV, hepatitis C virus; HIV, human immunodeficiency virus; SHA, severe hemophilia A; SHL, standard half‐life formulation.

^a^ Most recent year of data considered for each patient was defined from the most recent encounter.

^b^ Estimates mean treatment costs based on dosing, treatment frequency, and discontinuation observed in ATHN adult SHA population and assumed prices based on WAC prices.

## 4 DISCUSSION

The US hemophilia population has been clinically described as part of the centers for disease control and prevention (CDC) Universal Data Collection projects; however, recent clinical descriptions of the SHA population are more limited [16, 24, 32, 33]. The ATHNdataset is a large and primary source of clinical data on patients promoting better understanding of the burden of SHA. The present study summarizes the current sociodemographic and clinical characteristics, HTC‐related HRU, and HA treatments and projected costs among adult PwSHA, including among subpopulations of adult PwSHA.

This study describes current clinical management of the adult hemophilia population reflecting hemophilia A management with the use of prophylaxis and comprehensive care at HTCs [10, 14], while additionally highlighting changes in the population that should be considered as precision medicine is integrated into clinical practice [34]. Overall, the US adult SHA population receiving care management and HA‐related treatments at ATHN‐affiliated HTCs is diverse and broadly distributed across age, geographic region, race, ethnicity, and educational level. The population is generally distributed across ages, with the exception of the population with transfusion‐transmitted infections who tended to be older. This trend reflects the contamination of factor supply from the 1970s to the mid‐1980s, as well as modern blood screening practices that have dramatically reduced such errors since 1987 [35, 36], although not all infections necessarily occurred as a result of contamination of factor supply. This population will likely require focused care over the next decades to not only address these transfusion‐transmitted infections, but also age‐related clinical manifestations of hemophilia, such as chronic arthropathy and diseases of aging such as cardiovascular disease and cancer [37, 38, 39]. As the hemophilia A population generally reach older age, and currently for the population with transfusion‐transmitted infections, hemophilia specialists and HTCs may require additional training and staffing to provide comprehensive care. Similar to the implications of age for targeted hemophilia management, the variability in education level across the SHA population should be considered during management as well. For example, tailoring communication to an individual's level of understanding has been shown to be effective in improving disease control [40].

Further refinement and personalization in hemophilia care management may help to improve some of the trends in HRU observed in the current study, particularly regarding HTC visits, completion of annual comprehensive care assessments, and utilization of prophylaxis therapy. Although at a population level, PwSHA on average had 2.8 HTC visits per year and approximately one comprehensive care visit per year, approximately 25% of PwSHA were not seen at an HTC during their most recent year of data, and 33% of PwSHA did not have a comprehensive care visit. This highlights the overall success of disease management actives at HTCs overall while also highlight the potential for improvement for individual PwSHA, particularly those who are not utilizing the HTC. Telephone contacts were only utilized in 20% of patients but may be an option to reach patients who are not being seen at the HTC annually. Relatedly, with the increased utilization of telemedicine and virtual appointments as the US healthcare system adapted to the COVID‐19 pandemic, these virtual HTC visits may be another mechanism to reach PwSHA who are infrequently engaged with their HTC. The management of PwHA via telemedicine both before and during the pandemic has been assessed, finding that the disease is well suited for the benefits of telemedicine [41, 42]. Personalization in management may also help to ensure that PwSHA are treated with appropriate hemostatic treatments and reduce the utilization of on‐demand FVIII treatment regimens, which were still being utilized in 8% of PwSHA overall, including more than 10% of PwSHA and transfusion‐transmitted infections, rather than prophylaxis, which is the standard of care for all patients with severe hemophilia [10] and has been demonstrated to reduce bleeding events and HRU [15, 43]. Understanding individual patient factors in treatment decisions will likely be increasingly important as additional hemostatic treatment options are introduced.

One area of management where individualization already occurs is in prophylaxis regimens, as prophylaxis aims to take into considerations such as bleeding phenotype, joint status, patient preferences, and individual pharmacokinetics [10]. Due to this personalization, there has been limited description of real‐world prophylaxis regimens currently utilized in the US, likely related to limitations of available datasets, which is a strength of the ATHNdataset. Detailed, accurate dosing information is not generally available from alternative data sources such as administrative claims analysis or patient surveys. Additionally, clinical trial‐based reports do not accurately reflect typical dosing of prophylactic factor products commonly used in the US today as clinical practice has shifted from achieving a 1% trough level to adjusting dosing and dosing intervals sufficiently to prevent spontaneous and breakthrough bleeding and hemarthrosis [10]. Therefore, this analysis described current real‐world dosing with prophylaxis regimens in the USA. For example, the mean doses for SHL (40.3 IU/kg, every 2.6 days, equivalent of 108.5 IU/kg/week) and EHL (50.2 IU/kg, every 3.6 days, equivalent of 97.6 IU/kg/week) for PwSHA without inhibitors. These estimates are overall consistent with the limited previous description of prophylaxis regimens based on the ATHNdataset in the literature [16], as well as economic modeling inputs utilized by Institute for Clinical and Economic Review [44] where ATHN data were the primary source for estimating real world dosing. The absolute difference in each dosing level may be attributable to the set of specific factor products underlying the different analyses.

Precision medicine and consideration for individual factors in management are key to not only maximize patient outcomes but also optimize healthcare spending, as this analysis additionally highlights economic burden for PwSHA. For the overall cohort and each of the patient subpopulations, the projected mean annual treatment costs for EHL products ranged from $893,609 to $934,301, $798,700 to $930,812 for SHL products and $613,220 to $801,061 for plasma‐derived products. Previous analyses of HA‐related costs have identified that treatments account for the vast majority of costs [20]. These estimates of the economic burden of hemostatic treatment build upon those previously estimated by Croteau et al. (also based on ATHNdataset data), which projected the annual cost of prophylaxis to cost $753,480 with EHL and $690,144 with SHL [16]. Our estimates refine the estimates from Croteau et al. with updated data, as well as by utilizing reported individual patient dose, dose frequency, and weight along with product specific WAC prices. The previous analysis utilized median dosages, frequencies, and pricing from a representative EHL or SHL product.

The costs of treatment reported in the study are on the higher end compared to other reports assessing administrative claims data of similar study populations [20]. Differences in study time period, cohort definition, prophylactic treatments included, and type of cost reported (actual payer allowable payment vs. cost derived based on dosing information and unit price) may explain cost differences. Cost ranges from this study for PwSHA and inhibitors are similar to estimates from a study by Zhou et al., which reported that the presence of inhibitors added more than $800,000 per year to the treatment of a patient with HA [17, 45].

This study benefits from several strengths related to the ATHNdataset. First, the large sample of patients provides a current snapshot of the ATHN‐treated SHA patient population including regarding clinical characteristics that are not collected in other datasets, as well as describing subsets of the population. Additionally, the geographic reach of the ATHN‐affiliated HTC network and broad inclusion criteria for the analysis maximize generalizability of the findings to US hemophilia A management of PwSHA. However, the presented analyses are not without limitations. First, only data entered into the ATHNdataset were available for analysis; healthcare services received by patients through other medical providers were not captured. Observed HRU and projected costs are HA‐specific estimates incurred only at ATHN‐affiliated HTCs and may not reflect HRU provided by other healthcare providers. Additionally, the ATHNdataset is derived from data recorded in the EMR at ATHN‐affiliated HTCs. Therefore, the recording of specific variables may be better than others as highlighted by the different amounts of missing data in our analysis. For the observed data, missing data for the variables of interest were described as part of the analysis, and some variables (i.e., age at first bleed, age at first FVIII treatment) had a high proportion of patients with missing data. Diagnoses that may have occurred outside of the HTC, such as arthropathy, and HRU not occurring at the HTC, such as ED visits and hospitalizations, are likely under‐recorded in the ATHNdataset. Variation may also exist between HTC in the definition of data that is entered into the ATHNdataset, such as “telephone contacts,” which may be defined as any contact at some HTC and only contacts regarding a specific issues (e.g. excluding scheduling) at other HTCs. Second, although estimates are representative of clinical practice at ATHN‐affiliated HTCs, estimates may not be generalizable to non‐ATHN‐affiliated HTCs if the clinical practice patterns for ATHN‐affiliated versus unaffiliated medical providers treating PwSHA differ. Finally, costs were estimated using prescription data and WAC prices, which may not reflect the costs paid by payers after manufacturer rebates and/or patient cost sharing. For nonfactor product treatments (e.g., emicizumab), the mean dose and dose frequency reported in this study may have combined the loading and maintenance phases; overall, observed data are consistent with the indicated dose and frequencies in the product label.

## 5 CONCLUSION

This study describes the adult US SHA population, along with subpopulations, characterizing HTC‐related HRU and treatments as well as projections of economic burden associated with standard of care therapies. The study also highlights considerations when implementing precision medicine approaches, such as centralizing care for conditions of aging at the HTC, tailoring communication to the patient, and ensuring that patients have a comprehensive care visit each year. Implementation of precision approaches may be additionally supported by developing strategies for subgroups of patients with common characteristics, such as comorbid transfusion‐transmitted infections, arthropathy, and/or history of inhibitors. This study additionally highlighted the economic burden of current hemophilia treatment, further emphasizing the importance of tailored disease management in SHA.

## CONFLICT OF INTEREST

This study was funded by BioMarin. Hinds, Chen, and Solari are or were employees of BioMarin and own stock/stock options at the time of the analysis. Solari is currently an employee of Spark Therapeutics. Cheng, He, and Recht are, or were at the time of this study, employees of American Thrombosis and Hemostasis Network (ATHN), which has received ATHNdataset licensing and other fees from BioMarin.  Research funding to Dr. Recht's employers has come from Bayer, BioMarin, CSL Behring, Genentech, Grifols, Hema Biologics, LFB, Novo Nordisk, Octapharma, Pfizer, Sanofi, Spark, Takeda, and uniQure.  Dr. Recht has worked as a consultant for Catalyst Biosciences, CSL Behring, Genentech, Hema Biologics, Kedrion, Novo Nordisk, Pfizer, Sanofi, Takeda, and uniQure.  Dr. Recht sits on the board of directors of the Foundation for Women and Girls with Blood Disorders and Partners in Bleeding Disorders.  Dr. Recht is an employee of the American Thrombosis and Hemostasis Network and Oregon Health and Science University.

## ETHICS STATEMENT

The ATHNdataset is a certified, de‐identified dataset. The ATHNdataset has undergone ethics review and was deemed non‐human subjects research. Patient participation in the ATHNdataset is voluntary.
